# Autonomous Spiral Motion by a Small-Type Robot on an Obstacle-Available Surface

**DOI:** 10.3390/mi12040375

**Published:** 2021-04-01

**Authors:** Shinya Tokunaga, Chinthaka Premachandra, H. Waruna H. Premachandra, Hiroharu Kawanaka, Sagara Sumathipala, B. S. Sudantha

**Affiliations:** 1 Department of Electronic Engineering, School of Engineering, Shibaura Institute of Technology, Tokyo 135-8548, Japan; AG14074@shibaura-it.ac.jp; 2 Department of Electronic Engineering, School of Engineering/Graduate School of Engineering and Science, Shibaura Institute of Technology, Tokyo 135-8548, Japan; 3 ICT Center, Wayamba University of Sri Lanka, Gonawila 60170, Sri Lanka; warunaprema@yahoo.com; 4 Department of Electrical and Electronic Engineering, Graduate School of Engineering, Mie University, Mie 514-8507, Japan; kawanaka@elec.mie-u.ac.jp; 5 Faculty of Information Technology, University of Moratwua, Moratuwa 10400, Sri Lanka; sagarasns@gmail.com (S.S.); bh.sudantha@gmail.com (B.S.S.)

**Keywords:** spiral robot motion, fall prevention, obstacle avoidance, sensor fusion, motion analysis

## Abstract

Several robot-related studies have been conducted in recent years; however, studies on the autonomous travel of small mobile robots in small spaces are lacking. In this study, we investigate the development of autonomous travel for small robots that need to travel and cover the entire smooth surface, such as those employed for cleaning tables or solar panels. We consider an obstacle-available surface and target this travel on it by proposing a spiral motion method. To achieve the spiral motion, we focus on developing autonomous avoidance of obstacles, return to original path, and fall prevention when robots traverse a surface. The development of regular travel by a robot without an encoder is an important feature of this study. The traveled distance was measured using the traveling time. We achieved spiral motion by analyzing the data from multiple small sensors installed on the robot by introducing a new attitude-control method, and we ensured that the robot returned to the original spiral path autonomously after avoiding obstacles and without falling over the edge of the surface.

## 1. Introduction

With the evolution of computers and related technologies, several studies have been conducted on robots that can perform tasks deemed too risky or difficult for humans. To that end, the focus has been on developing wheeled robots [1,2,3,4,5,6,7,8,9], flying robots [10,11,12,13,14,15,16,17,18], and snakelike robots [19,20,21,22,23,24,25,26]. Many recent studies focus on developing not only robots that perform risky or difficult tasks for humans, but also production robots in workplaces that face labor shortages [27,28,29,30], cleaning robots to support housework to meet the demands of our busy lives [31,32,33,34,35], and robots to support the medical sector. Besides fixed robots (e.g., arm robots), most robots move and perform tasks in a wide range of environments; if robots have a wide range of movement, their large size does not constitute a problem. For example, the currently available home-cleaning robots are of a certain size, and because they are equipped with several high-performance functions, they can efficiently perform cleaning tasks, including smooth travel over smooth surfaces. In cleaning robots, path planning is very important. There are many studies in the literature regarding the path planning and autonomous movement of robots in comparatively larger indoor and outdoor areas [36,37,38,39]. Some of them have been targeted to achieve smooth movement within obstacle-available environments [40,41,42]. We experimentally found that applying these methods to small-type robots to achieve certain movement on a space like a table or a solar panel is difficult, since the computational capacity of a small-type robot is limited. In this paper, we mainly address this problem by making an appropriate problem setting and studying the problem with a small-type robot as mentioned below.

The motivation of this work is to achieve spiral travel of a small-type mobile robot to touch the maximum area of an obstacle-available surface (like a table), only with on-board lightweight hardware. Here, we investigate smooth travel for robots that can perform tasks such as cleaning tables or detecting cracks in solar panels. If the area of activity of the robot is small, the robot itself should be small. Therefore, in this study, we consider robots with a 10 cm × 10 cm footprint as small robots. We study regular travel on surfaces in small spaces as the basic technology for such robots to perform tasks such as cleaning tables or solar panels, and detecting defects in solar panels.

**Figure 1:**
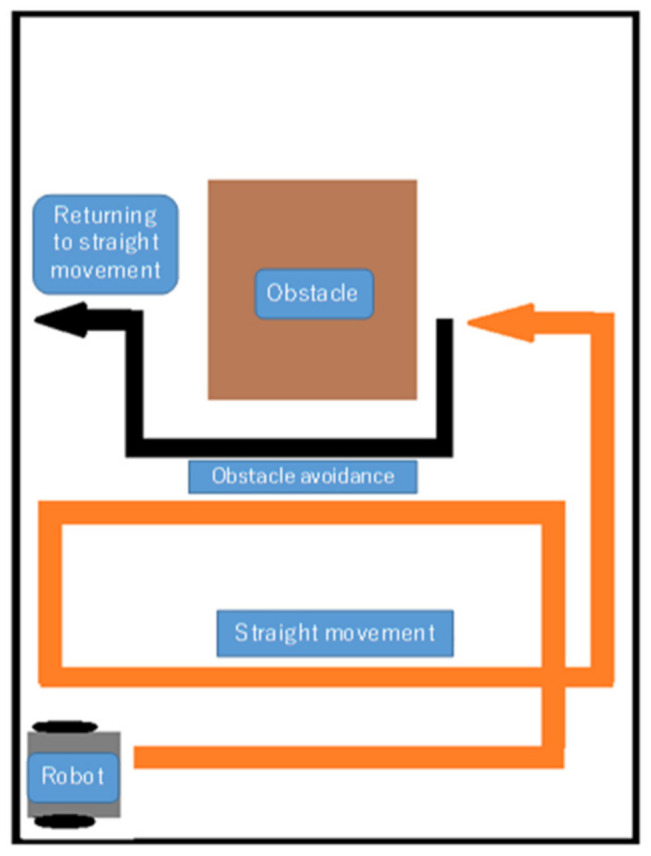
Robot movement to touch the majority of the moving surface.

Further, we study robot operation under the scenario in which an obstacle is present during travel, which the robot should avoid, and then return to its regular travel path autonomously. Figure 1 shows the specific movement that is considered in this study. In particular, this movement is a spiral movement that combines straight motion and U-turns. We believe that this movement will enable regular travel while covering most of the smooth surface, thereby allowing efficient performance in cleaning tasks.

There are previous studies that investigate regular travel [43,44,45,46]; however, they focus on simulations and not actual robot operations. The distance traveled by a mobile robot is conventionally measured using an embedded encoder [6,7]. The robot used in this study is not equipped with an encoder; we equipped a compact crawler robot with small hardware. The traveled distance was measured using the traveling time. To develop a small-type robot for which compactness is required, this kind of travel-time-based distance calculation can be applied. The development of regular travel, as shown in Figure 1, by a robot without an encoder is an important feature of this study. In this paper, we introduce a new attitude-control method. In this method, we obtain Euler angles from the onboard magnetic-field and acceleration sensors, and then convert them into quaternions and perform attitude control by updating the quaternions obtained from the onboard gyroscope sensor values. This is also one of the major contributions of this work. We achieve regular travel as shown in Figure 1, by analyzing the data from multiple small sensors installed on the robot, and we ensure that the robot returns to the original path autonomously after avoiding obstacles and without falling over the surface. This is a unique motion generation of this study.

We manufactured the robot described above and achieved the regular travel and fall prevention for this robot. All the processing that is necessary to achieve desirable autonomous robot motion is implemented by an onboard compact single-board microcontroller.

The remainder of this manuscript is structured as follows. Section 2 describes the hardware structure of the robot developed to carry out this work. Section 3 presents the proposed robot’s attitude-control method, and Section 4 describes method for fall prevention and obstacle avoidance. Section 5 presents and discusses the experimental verification of this work in detail. Finally, Section 6 concludes the paper.

## 2. Hardware of Developed Robot

### 2.1. Hardware Installed on the Robot

**Figure 2:**
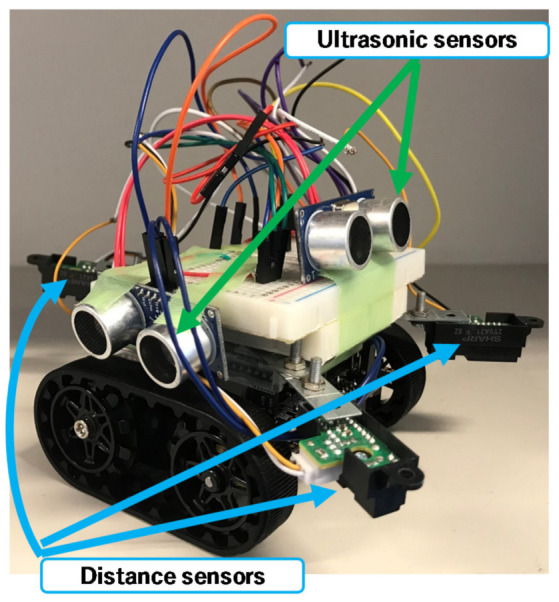
The robot developed to carry out this work.

The small robot designed in this study was created by fitting a commercially available Zumo robot—a crawler robot with an attitude and heading reference system (AHRS)—with new sensors, a single-board microcontroller, and additional small sensors. As shown in Figure 2, the robot was equipped with two additional ultrasonic sensors (front and side) and three distance sensors (GP2Y0A21YK; two on the front and one on the back). The system processes and robot attitude control were performed using the installed single-board microcontroller (Arduino UNO).

### 2.2. Hardware Roles

Table 1 lists the installed hardware and the original hardware of the Zumo robot, including their functions.

**Figure 3:**
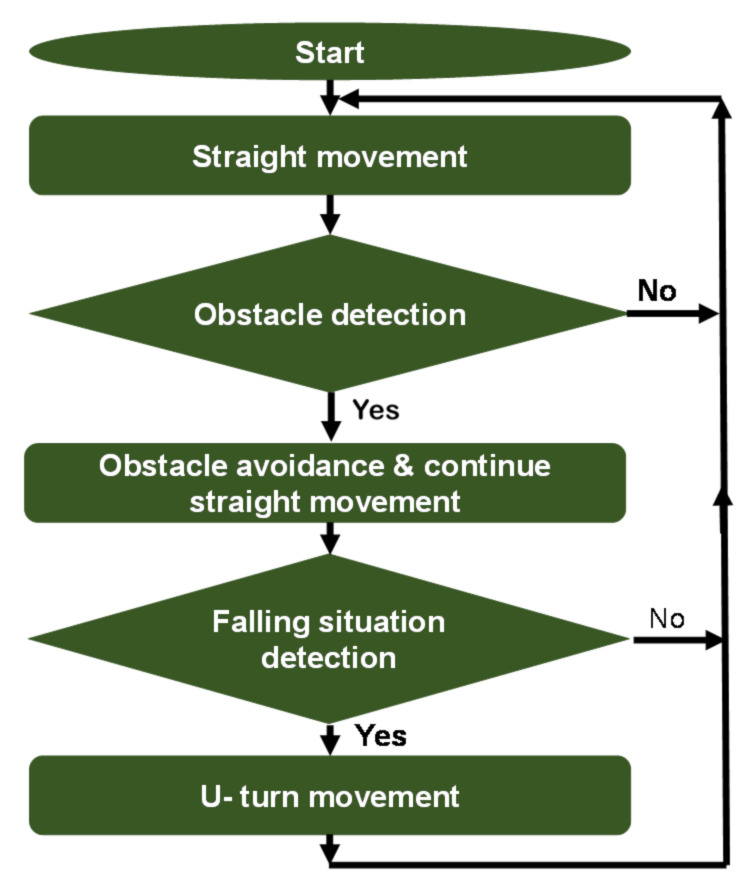
Flowchart of the robot’s movement generation.

The AHRS provides *z*-axis orientation output with the embedded gyroscope, acceleration, and magnetic field sensors. The main processing flow of achieving autonomous travel of the robot is shown in Figure 3. At the beginning, the robot starts traveling with a straight movement. If the robot finds the obstacle, it stops and begins the obstacle avoidance. The obstacle avoidance is done based on the outputs from the two onboard ultrasonic sensors. After avoiding the obstacle, the robot returns to the original movement and continues traveling. When the robot comes to the edge of the traveling space (table), falling prevention is conducted based on the outputs from the distance sensors. The falling situation is determined by using a predefined threshold value. After the falling prevention, the robot makes a U-turn and original spiral travel is conducted. During the U-turn and other movements, robot attitude control is conducted using the AHRS, as detailed in the next section.

## 3. Robot Attitude Control

In this study, regular travel refers to spiral motion. To ensure that the robot moved such that it covered the entire surface, we established a regular travel route where the traveled path is duplicated (Figure 1). Obstacle avoidance and robot attitude control were necessary to prevent the robot from falling off the table during motion.

### 3.1. Control Using AHRS

**Figure 4:**
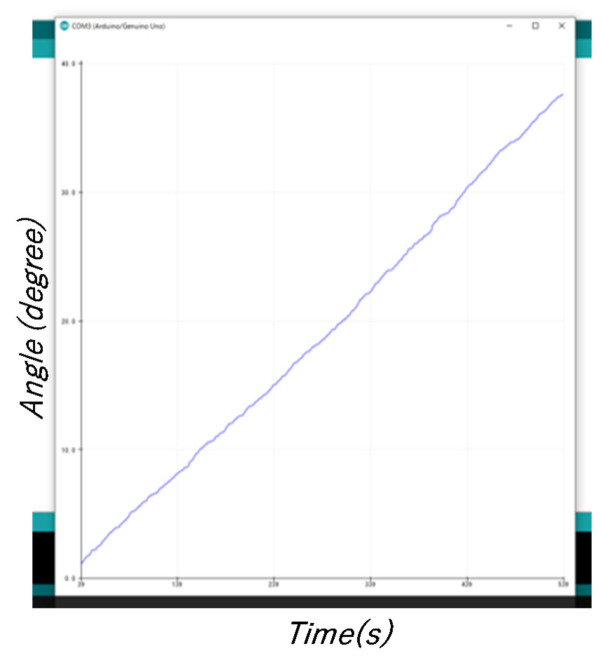
Angle detection only using a gyroscope sensor (when the robot was stationary).

The rotational yaw angle, which uses the Z axis of the robot (vertical axis of the object) as the rotational axis, is the parameter used for control; the attitude of the robot is controlled using AHRS. Angular velocity is detected through the gyroscope sensor, and the angle is obtained via integration (Figure 4).

The limitation of using only a gyroscope sensor to obtain the angle is that an angle is obtained even if there is no robot rotation (drift phenomenon). Therefore, it is difficult to achieve attitude control using only gyroscope sensors, and thus, we studied attitude control with AHRS.

(1) $$\theta =\theta_{t-1}+\omega \cdot dt$$

where  θ is the Euler angle, $\theta_{t-1}$ is the previous Euler angle, ω is the angular velocity, and *dt* is the integral time.

The angles expressed in Equation (1) are Euler angles that denote the respective degrees of rotation in relation to the three axes. In the Euler angle representation, results vary based on the order of rotation. Therefore, we used quaternions to obtain the angles easily. We obtained Euler angles from the magnetic field and acceleration sensors, and then converted them into quaternions and performed attitude control by updating the quaternions obtained from the gyroscope sensor values [47]. These steps are outlined below.

**Figure 5:**
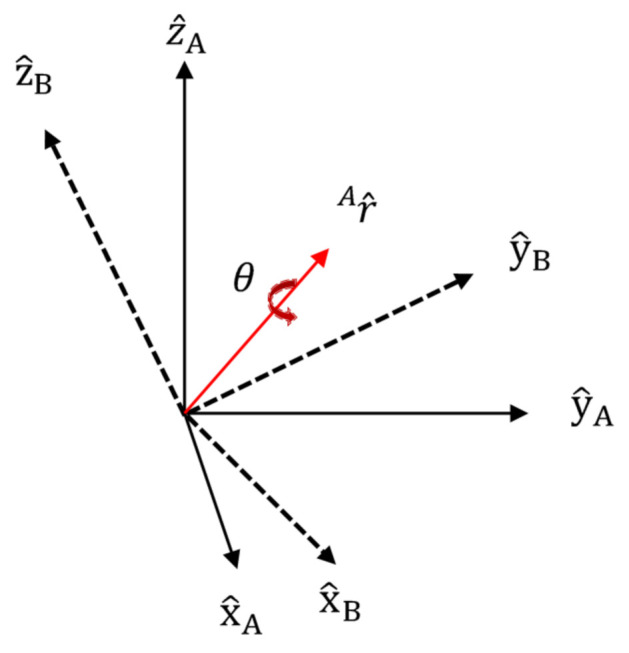
Quaternion representation.

First, we describe quaternions. Quaternions can be used to represent arbitrary angles to axes with arbitrary orientations. Figure 5 shows a quaternion representation. Since a vector designated as an axis must be normalized, an adjustment is performed. Rotation of angle A in relation to arbitrary orientation B in a 3D space can be represented as summarized below.

In Figure 5, ${\hat{x}}_{A},{\hat{y}}_{A},and\;{\hat{z}}_{A}$ are normalized coordinate axes in relation to arbitrary orientation A; ${\hat{x}}_{B},{\hat{y}}_{B},and\;{\hat{z}}_{B}$ are normalized coordinate axes in relation to arbitrary orientation B; r^ A is the vector in relation to arbitrary orientation A; and θ is the angle. The unit matrix for A in relation to orientation B, q,^BA is shown in Equation (2). Here, $r_{x},r_{y},r_{z}$ are the vectors for each element.

(2) q^BA=[q1 q2 q3 q4]=[cosθ2−rxsinθ2−rysinθ2−rzsinθ2]

The values obtained from AHRS were Euler angles. Quaternions require fewer computations than Euler angles, and therefore, we converted them into quaternions for the computations and converted them back into Euler angles later. For the conversions, we used Equations (3)–(5).

(3) $$q=\left[\begin{array}{c} cos\left(\frac{\varphi }{2}\right)cos\left(\frac{\theta }{2}\right)cos\left(\frac{\phi }{2}\right)+sin\left(\frac{\phi }{2}\right)sin\left(\frac{\theta }{2}\right)sin\left(\frac{\varphi }{2}\right)\\ sin\left(\frac{\varphi }{2}\right)cos\left(\frac{\theta }{2}\right)cos\left(\frac{\phi }{2}\right)-cos\left(\frac{\phi }{2}\right)sin\left(\frac{\theta }{2}\right)sin\left(\frac{\varphi }{2}\right)\\ cos\left(\frac{\varphi }{2}\right)sin\left(\frac{\theta }{2}\right)cos\left(\frac{\phi }{2}\right)+sin\left(\frac{\phi }{2}\right)cos\left(\frac{\theta }{2}\right)sin\left(\frac{\varphi }{2}\right)\\ cos\left(\frac{\varphi }{2}\right)cos\left(\frac{\theta }{2}\right)sin\left(\frac{\phi }{2}\right)-sin\left(\frac{\phi }{2}\right)sin\left(\frac{\theta }{2}\right)cos\left(\frac{\varphi }{2}\right) \end{array}\right]$$

where θ,ϕ,φ are the angles around axes x,y,and z, respectively.

(4) RBA=2q12−1+2q222q2q3+q1q42q2q4−q1q32q2q3−q1q42q12−1+2q322q3q4+q1q22q2q4+q1q32q3q4−q1q22q12−1+2q42,

where $q_{1},q_{2},q_{3},q_{4}$ are quaternion elements and RBA is the Euler angle of A in relation to orientation B.

To determine the degree of rotation in relation to the arbitrary axes, we needed to update the rotation in relation to the arbitrary axis and the three axes based on the gyroscope sensor values. The quaternions were updated based on gyroscope sensor values given by Equation (5).

(5) $$q=\left[\begin{array}{c} q_{1}\\ q_{2}\\ q_{3}\\ q_{4} \end{array}\right]\;\;=0.5\times \mathrm{dt}\left[\begin{array}{c} -(\omega_{x}\times q_{b}+\omega_{y}\times q_{c}+\omega_{z}\times q_{4})\\ \omega_{x}\times q_{a}-\omega_{y}\times q_{4}+\omega_{z}\times q_{c}\\ \omega_{x}\times q_{4}+\omega_{y}\times q_{a}-\omega_{z}\times q_{b}\\ \omega_{y}\times q_{b}-\omega_{x}\times q_{c}+\omega_{z}\times q_{a} \end{array}\right]\;$$

where $q_{a},q_{b},\;and\;q_{c}$ are the pre-updates $q_{1},q_{2},\;and\;q_{3},$ and $\omega_{x},\omega_{y}\;and\;\omega_{z}$ denote the angular velocity for each element.

### 3.2. Angle Detection by Each Sensor

The angles need to be calculated from the AHRS values. We studied the subsequent estimation of the current angles based on these respective angles. The AHRS values were used after offset correction and averaging.

(1) Gyroscope sensor

The gyroscope sensor measures the angular velocity around an axis. The sensor parameters are given by Equation (6), and angle vector is determined based on Equations (7) and (8).

(6) ω s=0ωxωyωz

where $\omega_{x},\omega_{y},and\;\omega_{z}$ denote the angular velocity for each element.

(7) q˙ESω,t=12q^ESest,t−1⊗ sωt

where q^ESest,t−1 is the previously normalized angle, ⊗ is the cross product, and ω st is the angular-velocity vector.

(8) qESω,t=q^ESest,t−1+q˙ESω,tΔt

where qESω,t is the angle vector.

As shown in Equations (7) and (8), the derivative for the quaternion is given by q˙ESω,t, and the current rotation angle is estimated through integration.

(2) Acceleration sensor

As there is no change in the acceleration angle, it is difficult to calculate the yaw angle. Further, if the sensor accelerates, it cannot be calculated. Each detected acceleration value must meet the conditions provided in Equation (9):

(9) $$\sqrt{a_{x}{}^{2}+a_{y}{}^{2}+a_{z}{}^{2}}=1G=9.8$$

where $a_{x},\;a_{y},\;\mathrm{and}\;a_{z}$ denote the acceleration of each axis. When this condition is met, the rotation pitch around axis x and the rotation roll around axis y can be expressed by Equations (10) and (11):

(10) $$\mathrm{pitch}=\tan^{-1}\frac{a_{x}}{\sqrt{a_{y}{}^{2}+a_{z}{}^{2}}}$$

(11) $$\mathrm{roll}=\tan^{-1}\frac{a_{y}}{a_{z}}$$

(3) Magnetic field sensor

If the roll and pitch information is known, the correct orientation can be estimated by the magnetic field sensor using these attitude data. When the magnetic field sensor output is $m_{x},m_{y},\;m_{z}$ and the roll and pitch angles obtained through Equations (10) and (11) are ϕ and φ, respectively, the obtainable yaw angle θ is as shown by Equation (12):

(12) $$\theta =\tan^{-1}\frac{m_{z}\sin\phi -m_{y}\cos\phi }{m_{x}\cos\varphi +m_{y}\sin\varphi \sin\phi +m_{z}\sin\varphi \cos\phi }$$

### 3.3. Estimation of Current Angle

The current angle is estimated from the angles obtained from each sensor. The methods to estimate the current angles when there is a slight change in the acceleration and a substantial change in the acceleration is shown below.

(1) Slight change in acceleration

The quaternion is updated based on the angular velocity obtained using the gyroscope sensor, where the amount of change is based on the angles obtained from the acceleration and magnetic field sensors (Equations (10)–(12)).

(2) Substantial change in acceleration

If there is change in relation to the initial acceleration vector after obtaining the angles from the acceleration and magnetic field sensors (Equations (10)–(12)), the displacement is corrected using the Madgwick filter [46] following Equation (13).

(13) qESest,t= q^ESest,t−1+q˙ESest,tΔt

where q˙ESest,t is the rate of change of orientation, qESest,t is the estimated angle, q^ESest,t−1 is the previous normalized angle, and Δt is the change over time. The correction method uses the gradient method based on the acceleration sensor vector. Thus, we can achieve attitude control for the traveling robot.

## 4. Fall Prevention and Obstacle Avoidance

Fall prevention is an important part of ensuring the robot’s regular travel on a flat surfaces such as a table or solar panel. Further, obstacle avoidance and the subsequent return to the regular travel path is important. For obstacle avoidance, the aim is to avoid an obstacle of a certain size such as a cardboard box, and subsequently, to return to the regular travel path.

### 4.1. Fall Prevention

**Figure 6:**
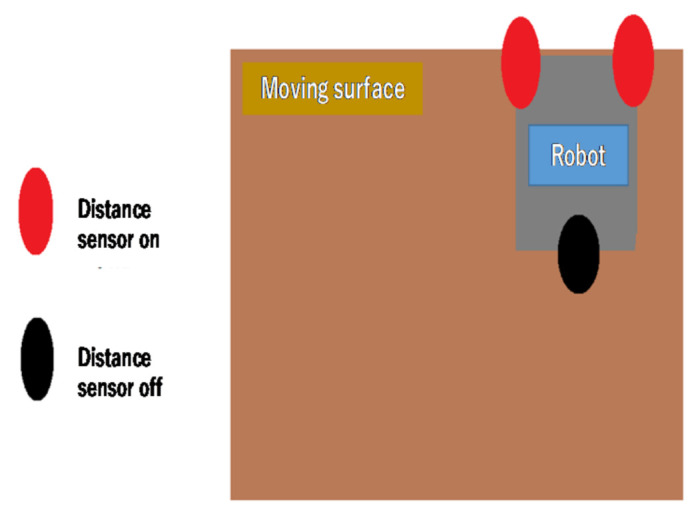
Distance sensor ON and OFF.

Fall prevention was achieved using the three distance sensors installed on the robot. Only one sensor was installed on the back of the robot because backward movement is performed by the robot in only a few limited scenarios. A threshold was set for the distance sensors to distinguish between the table (ON) and the other objects (OFF). When both sensors were switched to ON, the robot was stopped. Figure 6 shows the ON and OFF statuses of the sensors when the robot was stopped; these thresholds were determined experimentally for this study. After the robot temporarily stopped during its regular travel (Figure 1), it moved backward, made a U-turn, and continued its operation. On the other hand, if the one of two sensor pair was ON, the robot stopped the movement, and turning behavior was not done.

### 4.2. Obstacle Avoidance and Subsequent Return to Regular Travel

**Figure 7:**
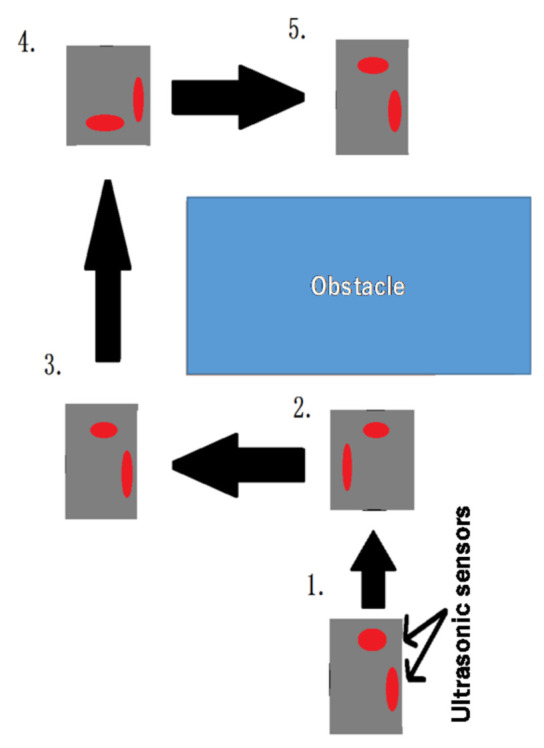
Returning to regular travel after avoiding obstacle.

We studied the autonomous obstacle detection of an object of certain size by a robot, its avoidance, and the subsequent return of the robot to its regular travel path. This operation was performed using ultrasonic sensors installed on the front and side, in the sequence shown in Figure 7. The steps are listed below.

1. The front sonic ultrasonic sensor detects an obstacle.
2. The robot performs a 90° left turn, switches on the ultrasonic sensor on the right side, and moves straight ahead until the right-hand ultrasonic sensor exceeds the threshold. Then, the time of the rectilinear movement is obtained, and using the acceleration sensor value, the current coordinates of the robot are calculated.
3. The robot performs a 90° right turn and moves straight ahead until the threshold of the right-hand ultrasonic sensor is exceeded. The time of the rectilinear movement is then obtained and the current coordinates of the robot are calculated following them. In the straight movement, the travel distance is calculated using the velocity×travel time, since in this study, the robot velocity is constant.
4. The robot performs a 90° right turn and moves straight ahead until its x coordinate is 0.5. The robot performs a 90° left turn and returns to its original regular travel. The coordinate calculations of the robot at this time are given by Equations (14) and (15):
  (14) $$x\;=\frac{\left(v\cdot time\right)}{1000}\cdot cos\left(\frac{\left(pi\cdot degree\right)}{180}\right)$$
  (15) $$y\;=\frac{\left(v\cdot time\right)}{1000}\cdot sin\left(\frac{\left(pi\cdot degree\right)}{180}\right)$$
  where *v* is robot velocity, time is the time of rectilinear movement, *pi* is the ratio of the circumference of the circle to its diameter, and degree is the rotation angle. These coordinates need to be adjusted in line with the actions of the robot; this is done only when required.

## 5. Experimental Verification

We conducted verification experiments for the proposed methods using an actual robot.

### 5.1. Experimental Environment

We tested the individual proposed methods and verified the system as a whole. Attitude control was verified by allowing the robot to travel on a table; we did this by making the robot perform a 90° rotation and checking its attitude-control performance.

**Figure 8:**
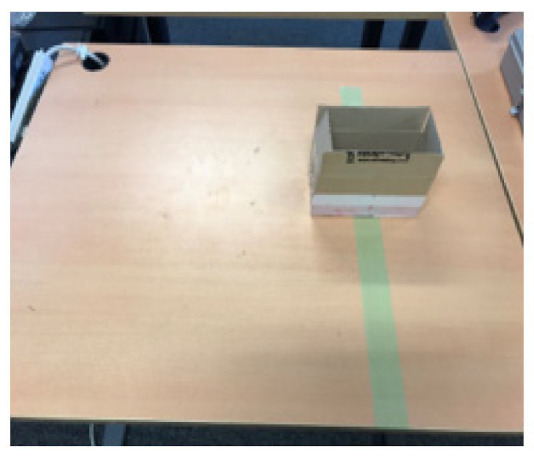
Example of a driving environment in the experiment.

For fall prevention, we made the robot move on a table and, as shown in Figure 8, checked that the robot stopped when reaching the edge of the table.

Obstacle avoidance and the subsequent return to the original travel path were verified by placing an obstacle on the table. The environment is shown in Figure 7. We verified whether the designed robot detected the obstacle and returned to its original travel path while avoiding the obstacle. Further, we verified the accuracy by placing the robot on a green line of table and checking whether the robot had returned to its location on its original travel path after avoiding the obstacle. In this work, the on-robot microcontroller (Arduino UNO) implemented all the robot motions.

### 5.2. Experimental Results

(1) Attitude control—verification results

**Figure 9:**
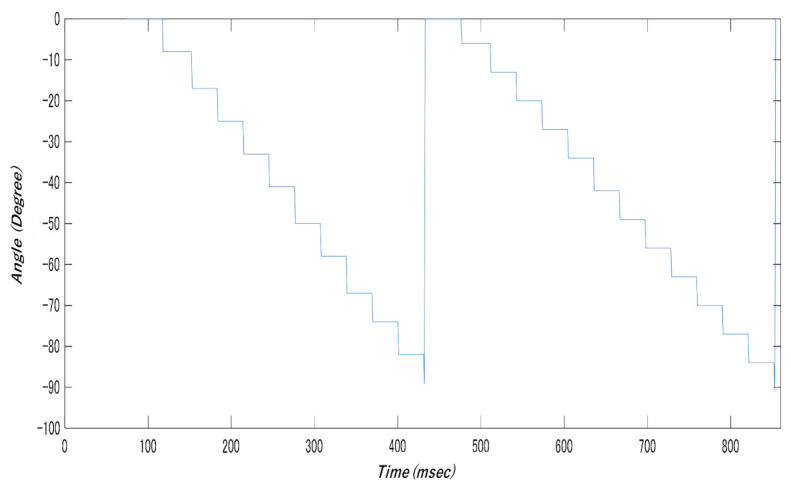
Attitude control test results (left-hand rotation).

**Figure 10:**
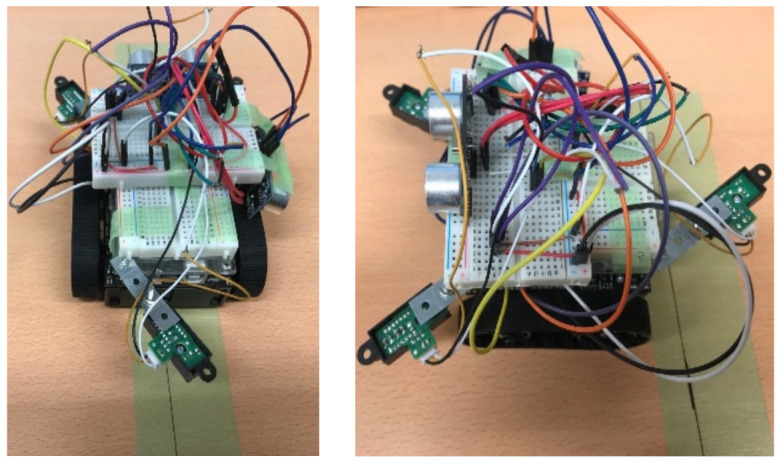
Robot prerotation (**left**) and postrotation (**right**).

The attitude control of the robot was checked using AHRS by verifying its accuracy when performing a 90° rotation. The operation involved repeated 90° rotations. Figure 9 shows rotations between 100 ms and 400 ms, and between 500 ms and 900 ms. Figure 10 shows the robot pre- and postrotation.

We performed multiple experiments in the same way, and the results of the attitude control performance checks were the same as in these experiments. There were few errors over multiple robot-rotation operations; however, sometimes errors gradually accumulated during travel.

(2) Fall prevention method—verification results

**Figure 11:**
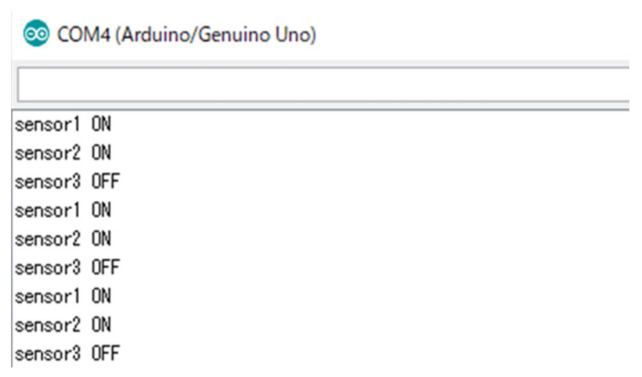
Output of distance sensors for fall prevention.

**Figure 12:**
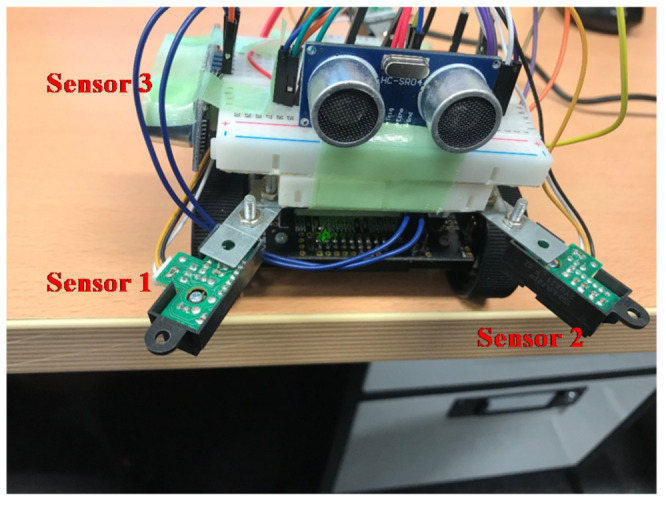
Robot at the time of the sensor output shown in Figure 11.

Since we focused on preventing the robot from falling forward, the results are for operation using the two front sensors. If sensors were placed above the table, they were considered OFF, and if not, they were considered ON. Figure 11 and Figure 12 show the actual performance results and robot position at the time. The experiment for this fall prevention technique was conducted 100 times, and it had a success rate of 99%, which is a good result. The failure case occurred due to a sudden slip of the robot.

(3) Obstacle avoidance and subsequent return to the original path—experiment

**Figure 13:**
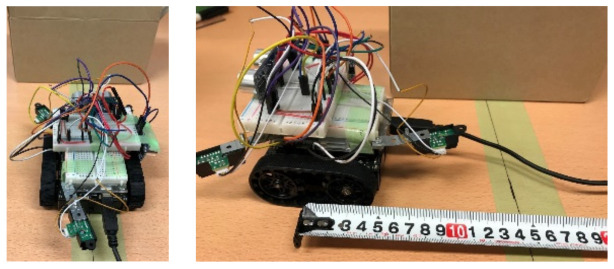
Robot detecting the obstacle (**left**); robot during obstacle avoidance (**right**).

**Figure 14:**
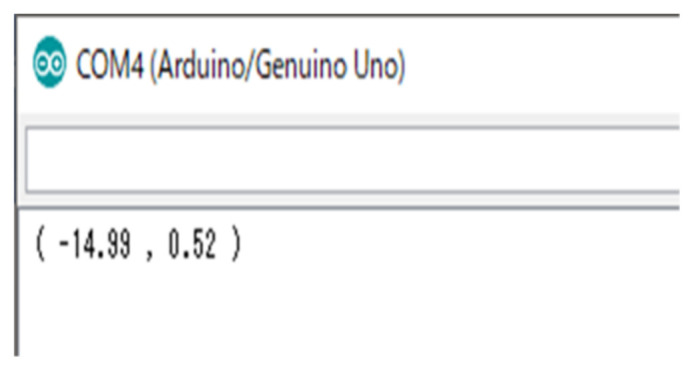
Robot’s own coordinate calculation results.

The robot’s operation involved rectilinear movement until the obstacle was found, and the avoidance of this obstacle so that the robot could return to its original travel path. Figure 13 shows the position of the robot when it detected the obstacle, and the random coordinate location of the robot during avoidance measured using a ruler. The measured coordinates of the robot when these measurements were taken are shown in Figure 14, and both were virtually consistent. Please see Supplementary Materials Video S1 for the obstacle avoidance performance.

(4) Overall regular travel—experimental results

**Figure 15:**
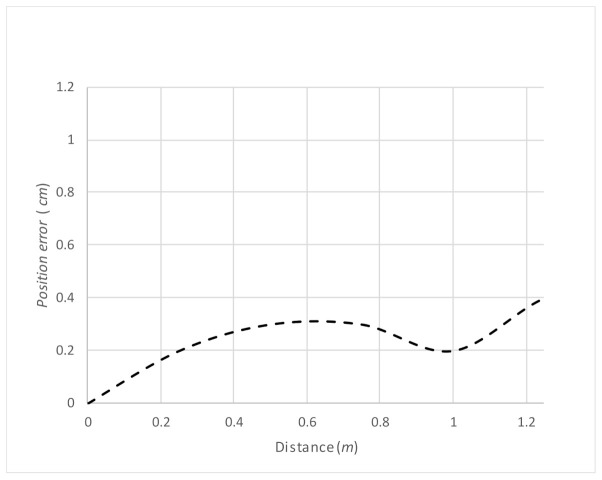
Errors in robot location during travel in the environment shown in Figure 8.

**Figure 16:**
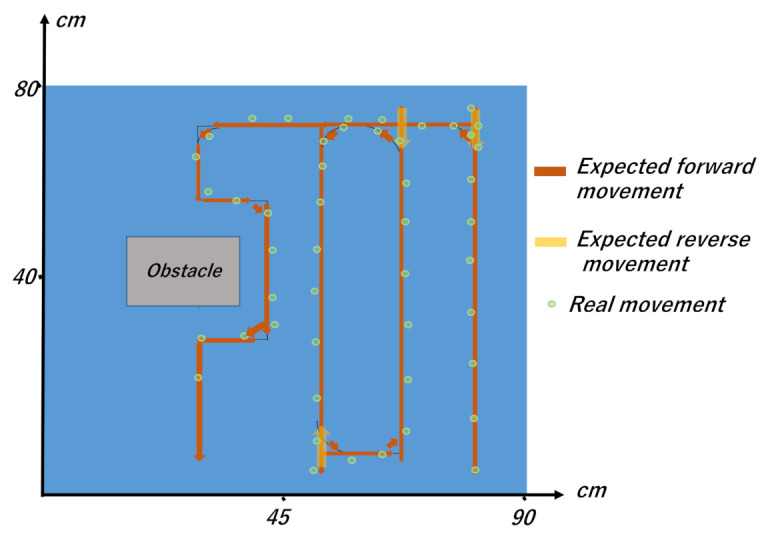
Trajectory of the expected travel and the real robot movement.

First, we conducted an experiment regarding the overall regular travel in the experimental environment shown in Figure 7. The experimental results of an overall travel of the robot on the table are demonstrated in Supplementary Materials Video S1. In the experiment shown in Supplementary Materials Video S1, the travel-path length and time were 465 cm and 75 s, respectively. For the same experiment, the measurement results for the errors in the location of the robot during travel are shown in Figure 15, and the trajectory of the expected travel and the real robot movement are shown in Figure 16. The errors were measured by drawing a straight line on the tape we applied on the table, and by having the robot travel autonomously on that line. The errors showed a tendency to increase depending on the traveled distance.

**Figure 17:**
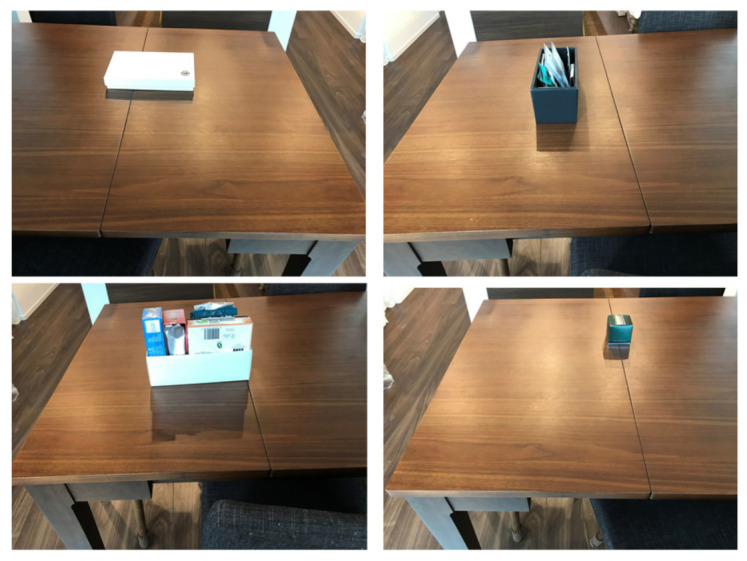
Different experimental environments.

Except for the environment shown in Figure 8, we further conducted experiments regarding the overall regular travel in different environments. Some of these environments are shown in Figure 17. Here, the experiments were conducted by changing the position of the obstacles as well.

In this paper, the evaluation was conducted based on the average position error during the motion. We separately measured the position error of straight motion and the position error of obstacle-avoidance motion. Table 2 illustrates the measured information. The average position error varied from 0.9 cm to 1.7 cm during the straight motion, while it varied from 1.3 cm to 2.1 cm during the obstacle avoidance motion. In addition to that, the average position error during the obstacle avoidance was always higher than that of straight motions. Here, during the obstacle avoidance, the robot made a motion following the information from the ultrasonic sensors. The main reason for the errors was that the ultrasonic sensors’ output included errors for certain levels.

### 5.3. Discussion Regarding Conventional Methods

As mentioned above, the target of this work was to achieve spiral travel of a small-type mobile robot while touching the maximum surface of an obstacle-available flat area (like a table), only with onboard lightweight hardware. There are many interesting studies that have been conducted for robot motion generation. Many of them have conducted only simulation experiments to make their evaluations [43,44,45]. However, in this work, we conducted all the experiments after manufacturing a real machine (mobile robot). Famous algorithms like the lawnmower algorithm could not be applied [48] in this work, since the implementation of the algorithm needs comparatively larger sensing devices like Lidar, which could not be installed on the robot of this work. On the other hand, some conventional studies have also been conducted to move the robot to a specific target while avoiding the obstacles with real machines [40,41]. The problem settings of those studies were different from this study. However, we could apply some of those works to achieve only straight motions in this study. Furthermore, many studies can be found in the literature for obstacle detection and avoidance [5,33,40,41,45,49,50,51,52]. However, in this study, the obstacle avoidance was done by keeping a constant distant from the obstacle, as shown in Figure 8. In addition to that, after avoiding the obstacle, the robot returned to the spiral motion and continued that motion. As mentioned above, in this work, the obstacle avoidance and returning to the original spiral motion was done based on onboard ultrasonic sensor outputs. According to our observations, we could not find any study in the literature that conducted a similar kind of obstacle-avoidance process. It is a unique motion generation of this study.

## 6. Conclusions

The study focused on developing a small robot that can travel on a flat surface, such as a table or solar panel, while covering most of the surface area. We equipped a robot with small sensors and a single-board microcontroller, and investigated the spiral travel path of the robot on an obstacle-available surface. We processed data obtained from these sensors and used the sensors to achieve attitude control and regular travel, obstacle avoidance, subsequent return to the original path, and fall prevention. In the experiments conducted so far, some level of error occurred in the case of regular travel, since the expected robot position did not coincide with the real robot position. We plan to work on correcting these errors in future studies. We successfully achieved obstacle avoidance for obstacles of a certain size and shape; we plan to study the avoidance of smaller obstacles in the future. In addition to that, achieving the spiral movement in moving obstacle-available environments would also be an interesting future stage.

## Figures and Tables

**Table 1:** Hardware roles.

Hardware	Role
AHRS	Robot attitude control
Ultrasonic sensor × 2	Front and side obstacle detection
Distance sensors × 3	Fall prevention; 2 on the front, 1 on the back
Arduino UNO	System processes and robot control

**Table 2:** Average position error.

Exp. Number	Average Position Error during Straight Motion (cm)	Average Position Error during Obstacle Avoidance (cm)
1	1.4	1.8
2	1.4	1.9
3	1.4	1.6
4	1.3	1.5
5	1.6	1.5
6	1.7	2.1
7	1.5	1.7
8	0.8	1.3
9	1.2	1.6
10	1.3	1.7
11	1.4	1.5
12	1.7	1.8
13	1.1	1.5
14	0.9	1.4
15	1.3	1.5

## Supplementary Materials

The following are available online at https://www.mdpi.com/article/10.3390/mi12040375/s1, Video S1: Video of the experimental results.
